# Supplementary material for: Patterns of Intron Gain and Loss in Fungi
Source: PLoS Biol. 2004 Nov 30;2(12):e422. doi: 10.1371/journal.pbio.0020422 (PMC532390; doi:10.1371/journal.pbio.0020422)
Supplement: Table S1 — Also available at http://genes.mit.edu/NielsenEtAl/. (4.3 MB ZIP). [file pbio.0020422.st001.zip › NielsenEtAl/html/492.html]

AN6869.1.NCU01348.1.MG10533.1.FG02206.1


```
 CLUSTAL W (1.82) Multiple Sequence Alignments - Introns Inserted


Sequence 1: NCU01348.1	392 aa
Sequence 2: FG02206.1	395 aa
Sequence 3: MG10533.1	395 aa
Sequence 4: AN6869.1	412 aa
Alignment Length: 417 aa
Number Identitical Residues: 277 aa
Alignment Score (without introns) 11955


MG10533.1 	-MVHASLLALGWAALASAHDTQKQVPLAGPLESVWYNTLPGDGGTQ0ADSVFSGITTFGR
NCU01348.1	-MLRPTFIASCLAAVAAAHGDHDQKVVAGPHQGLWYNTLPGDGGTQ0ADSVFSGISTFGR
FG02206.1 	-MLFKSFTFLSIASVAIAHGSHDQEPMEGPHQSLWYNTLPGDGGTQ0ADSVFSGISTFGR
AN6869.1  	MLVPVSLLALSLSSVAAAHSSH-QTPLTGPLQKLWYNTLPGDGGTQ0ADSVFSGISTFGR
          	 ::  ::     :::* **. : *  : ** : :************ ********:****

MG10533.1 	LPYAPCLKDSSVNYDIAFIG1APFDTGTSYRPGARFGPSGIRQGSRRLNLY2GGYNVPLA
NCU01348.1	LPYLPCLASKDINYDIAFIG1APFDTGTSYRPGARFGPSGIRQGSRRLNLY2GGYNVPLA
FG02206.1 	LPYQPCLGHNSIKYDIAFIG~APFDTGTSYRPGARFGPSGIRQGSRRLNLY2GGYNVPLA
AN6869.1  	LPYFPCLASEEERFDIAFIG~APFDTGTSYRPGARFGPSGIRQGSRRLNLY2GGYNVPLQ
          	*** ***  .. .:****** ****************************** ******* 

MG10533.1 	VNPFNSWARVLDCGDIPVTS2-------------------YDNTWALRQIEEGHHQILSR
NCU01348.1	TNPFNSWATIIDCGDIPVTS~-------------------YDNTFALSQIERGHHSILSR
FG02206.1 	TNPFNSWATVLDCGDIPVTS2-------------------YDNQFALQQIEEGHYSILSR
AN6869.1  	ANPFSNDLKVLDCGDIPVTS~YASLSPMSIPEDVCADCRRYDNAWAIQQIEEGHNSLLMR
          	.***..   ::*********  :: :. : ... .:..  *** :*: ***.** .:* *

MG10533.1 	APATNANKAGPALRSKTLPRVITLGGDHTITLPLLRSINRAYGPVSVIHFDSHL~DTW1K
NCU01348.1	KPTTDSALPGPSLQGKTLPRVITLGGDHTITLPLLRSINRSYGPVSVIHFDSHL~DSW~R
FG02206.1 	PPVTDADKLGPASKGRTLPRVITLGGDHTITLPLLRSINRAYGPVTVIHFDSHL~DTW1R
AN6869.1  	KPYTSANEYGLSRAGKTLPRIITLGGDHTITLPLLRSINKAYGPVTVIHFDSHL2DTW~K
          	 * *.:   * :  .:****:******************::****:******** *:* :

MG10533.1 	PKVFGGSPSEVASINHGTYFYHASMEGLLHNDTNIHAGIRTTLSGPSDYANDGYCGFEIV
NCU01348.1	PKVFGGSPSEVASVNHGTYFYHAAQEGLLRNDSNIHAGIRTTLSGPSGYENDGYCGFEIV
FG02206.1 	PKVFGGSPSEAASINHGTYFYHAAMEGLLVNDTNIHAGIRTTLSGPSDYDNDGYCGFEIV
AN6869.1  	PKVFGGSPSEVASINHGTYFYHAAMEGLLRNDTNIHAGIRTTLSGPSDYENDGYVGFEIV
          	**********.**:*********: **** **:**************.* **** *****

MG10533.1 	EAREIDTIGTTGIIKRIVERVGTK--NPVYLSIDIDTLDPAF~APATGTPETGGWSTREL
NCU01348.1	EAREIDTIGTEGIIKKIRERVGTE--KPVYLSLDIDTLDPAF1APATGTPETGGWSTREL
FG02206.1 	EAREIDTIGTTGIIEKILKRVGPD--RPVYLSIDIDTLDPAF~APATGTPETGGWSTREL
AN6869.1  	EAREIDTIGTEGIIKKIRDRVGNDPSTPIYLSIDIDTIDPAF~APATGTPETGGWSTREL
          	********** ***::* .*** ..: *:***:****:**** *****************

MG10533.1 	RTILRGLEDLNLIAADIVEVA0PAYDTNAEHTTMAAADVLYEVMSMMVKKGPLSNMIEEE
NCU01348.1	RTILRGLEGINIIAADIVEVA0PAYDTNAEHTTMAAADALYEIMSIMVKRGPLSLVSEP-
FG02206.1 	RTIIRGLESLNLIAADIVEVA0PAYDTNAEHTTMAAADVLYEVMSIMVKKGPLSRMMDGK
AN6869.1  	RTLIRGLDGLNLIGADIVEVA~PAYDTNAELTTMAAADVLYEVLTIMVKKGPLTVDERS-
          	**::***:.:*:*.******* ******** *******.***::::***:***:      

MG10533.1 	AIGDL
NCU01348.1	--HDL
FG02206.1 	LQEEL
AN6869.1  	---DL
          	   :*
```
